# Dancing with Hormones: A Current Perspective of Nitrate Signaling and Regulation in *Arabidopsis*

**DOI:** 10.3389/fpls.2017.01697

**Published:** 2017-09-28

**Authors:** Peizhu Guan

**Affiliations:** Section of Cell and Developmental Biology, Division of Biological Sciences, University of California, San Diego, La Jolla, CA, United States

**Keywords:** nitrate signaling, hormones, TOR signaling, cell cycle, root growth

## Abstract

In nature and agriculture, nitrate availability is a main environmental cue for plant growth, development and stress responses. Nitrate signaling and regulation are hence at the center of communications between plant intrinsic programs and the environment. It is also well known that endogenous phytohormones play numerous critical roles in integrating extrinsic cues and intrinsic responses, regulating and refining almost all aspects of plant growth, development and stress responses. Therefore, interaction between nitrate and phytohormones, such as auxins, cytokinins, abscisic acid, gibberellins, and ethylene, is prevalent. The growing evidence indicates that biosynthesis, de-conjugation, transport, and signaling of hormones are partly controlled by nitrate signaling. Recent advances with nitrate signaling and transcriptional regulation in *Arabidopsis* give rise to new paradigms. Given the comprehensive nitrate transport, sensing, signaling and regulations at the level of the cell and organism, nitrate itself is a local and long-distance signal molecule, conveying N status at the whole-plant level. A direct molecular link between nitrate signaling and cell cycle progression was revealed with TEOSINTE BRANCHED1/CYCLOIDEA/PROLIFERATING CELL FACTOR1-20 (TCP20) – NIN-LIKE PROTEIN 6/7 (NLP6/7) regulatory nexus. NLPs are key regulators of nitrogen responses in plants. TCPs function as the main regulators of plant morphology and architecture, with the emerging role as integrators of plant developmental responses to the environment. By analogy with auxin being proposed as a plant morphogen, nitrate may be an environmental morphogen. The morphogen-gradient-dependent and cell-autonomous mechanisms of nitrate signaling and regulation are an integral part of cell growth and cell identification. This is especially true in root meristem growth that is regulated by intertwined nitrate, phytohormones, and glucose-TOR signaling pathways. Furthermore, the nitrate transcriptional hierarchy is emerging. Nitrate regulators in primary nitrate signaling can individually and combinatorially control downstream transcriptional networks and hormonal pathways for signal propagation and amplification. Under the new paradigms, nitrate-induced hormone metabolism and signaling deserve fresh examination. The close interplay and convergent regulation of nitrate and hormonal signaling at morphological, physiological, and molecular levels have significant effects on important agronomic traits, especially nutrient-dependent adaptive root system growth and architecture.

## Introduction

As a constituent of amino acids and nucleotides, nitrogen (N) is an essential building block for all forms of life. Not surprisingly, the mineral nutrient needed in greatest abundance by plants is N (Crawford, 1995). N availability is crucial for plant anabolism and catabolism. Despite the abundance of N (78%) in the atmosphere, the availability of fixed N in Earth’s crust is scarce to such an extent that N is the quantitatively most limiting nutrient for plants (Vance, 2001; Miller and Cramer, 2004). In nature and agricultural systems, plants take up N mainly from soils in two forms, nitrate and ammonium, by roots during their postembryonic growth. Nitrate is the predominant form of N in aerobic soils where nitrification occurs rapidly (Crawford and Forde, 2002). As most soils on Earth are aerobic, nitrate is a primary N source and hence an essential nutrient for most plants.

Plants are sessile organisms that always face spatiotemporal fluctuations of nitrate concentrations in soil solution by up to four orders of magnitude due to leaching and microbial activity (Crawford, 1995; Vance, 2001; Miller and Cramer, 2004). In interacting with the environment, plants have evolved elaborate adaptive sensing, signaling and regulatory network in response to nitrate availability for survival, fitness and reproduction (Crawford, 1995; Wang et al., 2004, 2012; Remans et al., 2006a,b; Ho et al., 2009; Ruffel et al., 2011; Marchive et al., 2013; Guan et al., 2014, 2017; Vidal et al., 2015; Bellegarde et al., 2017; Liu et al., 2017). Nitrate is hence an essential nutrient as well as a crucial signal for plant growth, development, and stress responses.

Furthermore, nitrate and hormonal signaling and their interaction are of fundamental importance, underlying a plethora of plant physiological, morphological, and developmental processes in plants (Krouk et al., 2011; Nacry et al., 2013; Krapp, 2015; Krouk, 2016; O’Brien et al., 2016; Bellegarde et al., 2017). Much of our understanding of the process has been achieved so far by the molecular genetic studies using *Arabidopsis thaliana* as a model. The accumulating evidence indicates that biosynthesis, de-conjugation, degradation, transport, and signaling of hormones are partly controlled by nitrate signaling, so that the environmental and internal signaling pathways are seamlessly integrated.

Much of the literature on the interaction between nitrate and hormonal signaling pathways has been focused on hormonal control of nitrate metabolism and signaling (Kiba et al., 2010), while this review focuses more on the other side of the coin – nitrate signaling control of hormone metabolism and signaling (Krouk, 2016). The latest findings in nitrate research revealed novel molecular links between N and plant development (Ristova et al., 2016; Guan et al., 2017; Liu et al., 2017) shed new light on nitrate-hormone interconnections. In the context of agronomy, nitrate- and hormone-regulated lateral root (LR) growth and development are among the main determinants of root plasticity in response to nitrate availability and of nitrogen use efficiency (NUE) in crops. Therefore, the interplay and convergent regulation of nitrate and hormones in LR, which is highlighted in this review, is of agronomic importance.

## Nitrate Signaling, Uncoupled from Nitrate Metabolism, Acts at Local and Whole-Plant Levels

Nitrate is taken up by roots then transported into root and shoot cells mainly via the NITRATE TRANSPORTER 1 (NRT1) and NITRATE TRANSPORTER 2 (NRT2) family of nitrate transporters (Wang et al., 2012). Once inside the cells, nitrate is reduced to nitrite by NITRATE REDUCTASE (NR) in the cytosol. In *A. thaliana*, two NR enzymes, NIA1 and NIA2, are responsible for 10 and 90% of the total NR activity in seedlings, respectively (Cheng et al., 1988; Wilkinson and Crawford, 1991, 1993). Nitrite is then reduced to ammonium by NITRITE REDUCTASE (NiR) in plastids, where ammonium is in turn assimilated into glutamine (Gln) (Crawford, 1995). Notably, with external nitrate concentration increases, nitrate assimilation into amino acids in higher plants is increasingly achieved in shoots, which become the main sites of NR activity (Andrews, 1986). In addition to amino acids production, nitrate metabolism supports plant use of light, CO_2_ and water to produce sugars and organic acids. In spite of its importance, nitrate assimilation is energetically costly, demanding intensive use of adenosine triphosphate (ATP), reducing equivalents, and C skeletons (Nunes-Nesi et al., 2010). Therefore, nitrate assimilation is subject to restraint or stimulation by resource availability in the environment and the demands of plant growth and development.

Fundamentally, nitrate signaling is uncoupled from, but executes tight control over, nitrate metabolism (Wang et al., 2000, 2003, 2004, 2007, 2009; Scheible et al., 2004; Muños et al., 2004; Ho et al., 2009; Nunes-Nesi et al., 2010; Konishi and Yanagisawa, 2013; Marchive et al., 2013; Bouguyon et al., 2015; Guan et al., 2017; Liu et al., 2017). Most plants have been wired to perceive nitrate, but not its downstream metabolites, e.g., ammonium and Gln, as the principal source of N offered by the environment. Among the earliest and most convincing evidence is that revealed by transcriptome analysis in NR-null (*nia1 nia2*) mutants, numerous genes, including the key nitrate assimilatory genes, directly respond to nitrate independent of nitrate reduction (Wang et al., 2004). Hence, nitrate is a signal molecule of paramount importance, from stimulating germination, to sustaining substantial postembryonic growth, to controlling developmental phase transitions (Alboresi et al., 2005; Chopin et al., 2007; Fan et al., 2009; Nunes-Nesi et al., 2010; Vidal et al., 2014b; Yan et al., 2016; Guan et al., 2017; Liu et al., 2017). In response to soil nitrate availability, plants reprogram genome-wide short-term and long-term gene expression at the whole-plant level and promote adaptive regulation of organogenesis, involving root system architecture, root and shoot growth, leaf expansion, flowering time, stomata opening, defense responses, etc. (Forde, 2002; Walch-Liu et al., 2005; Krouk et al., 2010a; Castro-Marín et al., 2011; Ruffel et al., 2011; Fagard et al., 2014; Guan et al., 2014, 2017; Liu et al., 2017).

When exposed to nitrate, the first, and also one of the foremost nitrate responses in plants that has been extensively studied is the primary nitrate response (PNR) (Redinbaugh and Campbell, 1991; Wang et al., 2000, 2003, 2004, 2007; Scheible et al., 2004; Medici and Krouk, 2014; Ristova et al., 2016). The PNR is rapid, independent of *de novo* protein synthesis, and responsive to nitrate concentrations as low as 100 nM in roots and 250 μM in shoots of pre-starved *Arabidopsis* seedlings. The PNR affects the expression of 1,596 genes at the significance level in wild-type (WT) plants. Among those genes, 595 genes in both roots and shoots directly responded to nitrate, which is confirmed in the NR-null mutant (*nia1 nia2*) (Wang et al., 2004).

Indeed, the nitrate response is a whole-plant response and nitrate itself can function as both a local and long-distance systemic signal (Wang et al., 2004; Ruffel et al., 2011). The phenomenon was initially revealed by microarray analysis of nitrate-regulated gene expression in roots and shoots of the seedlings that were grown hydroponically. When treated with 0.25 mM nitrate for only 20 min, the roots have a much broader response than shoots in terms of the number of genes being affected (Wang et al., 2000). However, with sufficient nitrate concentrations and sufficient time (5 mM nitrate for 2 h) for nitrate transport facilitation (but not for reduction), the shoot genes in the NR-null mutant can be as responsive to nitrate as root genes, although the two groups of genes are still organ-specific (Wang et al., 2004). The PNR genes in roots and shoots are selectively targeted, with an overall concentration on energy and metabolism, including glycolysis and gluconeogenesis, amino acid metabolism, nitrogen and sulfur utilization, and transport facilitation (Wang et al., 2004); nevertheless, there are also a large group of genes for signaling and regulatory components intimately related to the two-component systems (TCS), calcium and sugar transport, auxin, cytokinins, and abscisic acid (ABA) metabolism and signaling, and so on (Wang et al., 2000, 2003, 2004; Ristova et al., 2016; Liu et al., 2017).

Nitrate signaling and regulation underlie the genome-wide expression reprogramming in nitrate responses, which leads to activation and adaptation of N-regulated metabolism and development. The process involves membrane and cytosol sensing, signal transduction, transcription factors (TFs), the interactions of TFs, and nitrate-responsive DNA regulatory elements (Ho et al., 2009; Wang et al., 2010; Guan et al., 2014, 2017; Liu et al., 2017). On the other hand, the transcriptome analysis revealed that numerous pathways and processes, particularly hormone signaling pathways, have interaction with and depend on nitrate signaling (Krouk, 2016; O’Brien et al., 2016; Ristova et al., 2016) for N status before making collective decisions in growth, development and stress responses.

## Nitrate Transport, Signaling and Regulation at a Glance

In plants, the first identified and characterized nitrate transporter is known as chlorate resistant 1 (CHL1) or AtNRT1.1 or AtNPF6.3 (Tsay et al., 1993). It is also the first plant member of NITRATE TRANSPORTER 1/PEPTIDE TRANSPORTER (NRT1/PTR) Family (also named NPF) discovered. The NRT1/PTR Family (NPF) comprises of membrane proteins ubiquitously found across all major kingdoms of life and sharing sequence homology. In bacteria, fungi, animals and plants, the family members were found to transport dipeptides (Leran et al., 2014; von Wittgenstein et al., 2014). In higher plants, there are at least four families of nitrate transporters. Besides NRT1/PTRs, the other three families are: NITRATE TRANSPORTER 2 (NRT2), CHLORIDE CHANNEL (CLC) a/b, and SLOW ANION CHANNEL-ASSOCIATED 1 HOMOLOG 3 (SLAH3) (Wang et al., 2012; Krapp et al., 2014).

**FIGURE 1:**
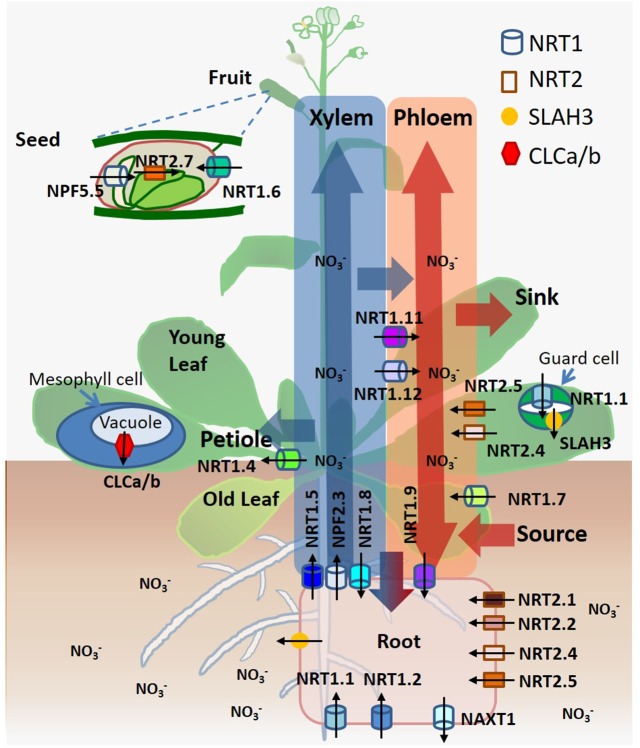
A summary of spatiotemporal functionality of nitrate transporters/channels and nitrate transport routes in *Arabidopsis*. Nitrate is taken up by roots, loaded/unloaded by xylem and phloem, and transported to leaves, shoots and seeds. Arrows indicate the directions of nitrate movement. Transporters and channels are depicted according to their localization.

The nitrate transporters contribute to numerous physiological functions involved in different stages and processes of nitrate distribution, assimilation, signaling, and osmotic regulation. They are individually critical, such as NRT1.1 (CHL1/NPF6.3), NRT1.2 (NPF4.6/AIT1), NRT2.1, NRT2.2, NRT2.4, and NRT2.5 in nitrate uptake from soil (Tsay et al., 1993; Huang et al., 1999; Cerezo et al., 2001; Kiba et al., 2012; Lezhneva et al., 2014); NAXT1 (NPF2.7) in nitrate efflux (Segonzac et al., 2007); NRT1.5 (NPF7.3), NRT1.8 (NPF7.2), and NRT1.9 (NPF2.9) in root-to-shoot xylem translocation, a primary route of long-distance nitrate transport driven by transpiration, which is accompanied by shoot-to-root xylem and phloem transport of nitrate (Lin et al., 2008; Li et al., 2010; Wang and Tsay, 2011); NRT1.7 (NPF2.13), NRT1.9 (NPF2.9), NRT2.4 and NRT2.5 in source-to-sink phloem remobilization, a secondary route of long-distance nitrate transport driven by osmotic gradients in both roots and shoots (Fan et al., 2009; Wang and Tsay, 2011; Kiba et al., 2012; Lezhneva et al., 2014); NRT1.4 (NPF6.2) in nitrate petiole storage (Chiu et al., 2004); CLCa/b in nitrate accumulation in vacuoles (De Angeli et al., 2006; von der Fecht-Bartenbach et al., 2010); NRT1.6 (NPF2.12) and NRT2.7 in nitrate accumulation in seeds (Chopin et al., 2007; Almagro et al., 2008); NRT1.1 (CHL1/NPF6.3) and SLAH3 in stomatal closure and opening (Guo et al., 2003; Geiger et al., 2011); NRT1.11 (NPF1.2) and NRT1.12 (NPF1.1) in xylem-to-phloem transfer for redistributing nitrate (Hsu and Tsay, 2013); NPF2.3 in nitrate translocation to shoots for acclimation to salt stress (Taochy et al., 2015); and NPF5.5 in embryo N accumulation (Leran et al., 2015) (**Figure 1**). All transport proteins are localized at the plasma membrane, except that NRT2.7 and CLCa/b are localized at the tonoplast (Wang et al., 2012; O’Brien et al., 2016).

As a result of dramatic fluctuations of nitrate concentrations in soil, plants have evolved two uptake systems: low-affinity transport system (LATS) for high external nitrate concentration (>0.5 mM) and high-affinity transport system (HATS) for low nitrate concentration (<0.5 mM), into which most nitrate transporters in roots and shoots can be categorized (Crawford and Glass, 1998; Forde, 2000; Miller et al., 2007). All known NPF transporters, except NRT1.1 (CHL1/NPF6.3), solely belong to LATS. Even though a majority of low- and high-affinity transporters are inducible, the two exceptions are NRT2.5 in HATS and NRT1.2 (NPF4.6/AIT1) in LATS, which are constitutive nitrate transporters (Huang et al., 1999; Lezhneva et al., 2014; Kotur and Glass, 2015). This gave rise to the four subsystems: the constitutive high-affinity system (cHATS), the inducible high-affinity system (iHATS), the constitutive low-affinity system (cLATS), and the inducible low-affinity system (iLATS) (Crawford and Glass, 1998; Forde, 2000; Tsay et al., 2007). Much attention has been given to the nitrate transporters that play crucial roles of mineral uptake in roots, the principal nutrient absorbing organs. In *Arabidopsis* roots, LATS involves NRT1.1 (CHL1/NPF6.3) and NRT1.2 (NPF4.6/AIT1) (Tsay et al., 1993; Huang et al., 1999), and HATS involves NRT1.1 (CHL1/NPF6.3), NRT2.1, NRT2.2, NRT2.4, and NRT2.5 (Wang et al., 1998; Liu et al., 1999; Cerezo et al., 2001; Li et al., 2007; Kiba et al., 2012; Lezhneva et al., 2014; Kotur and Glass, 2015). The NRT1s and NRT2s are both proton-coupled transporters. The interaction with NAR2 is critical for transport capacity of most high-affinity NRT2s in plants (Kotur et al., 2012; Kotur and Glass, 2015). When root epidermal cells are exposed to nitrate, the H^+^-ATPase in the plasma membrane pumps protons out of the cell, producing pH and electrical (ΔΨ) gradients, which potentially provides required energy to both LATS and HATS for co-transporting two or more protons per nitrate into the cell, a process also involving membrane depolarization. Both nitrate influx and efflux could be mediated by the proton-coupled mechanism (Crawford, 1995; Forde, 2000; Tsay et al., 2007; Wang et al., 2012).

Among the nitrate transporters so far characterized in *Arabidopsis*, NRT1.1 (CHL1/NPF6.3) is the only dual-affinity transporter (Wang et al., 1998; Liu et al., 1999; Liu and Tsay, 2003), although dual-affinity transport activity was also found in the potassium transporter AtKUP (Fu and Luan, 1998; Kim et al., 1998) and the nitrate transporter MtNRT1.3 (Morère-Le Paven et al., 2011). Moreover, NRT1.1 (CHL1/NPF6.3) mediates the expression of NRT2.1 and NRT3.1/ NAR2.1, depending on nitrate/ammonium concentrations. The process is the critical regulation of HATS, which is also under the feedback repression by N metabolites (Muños et al., 2004; Krouk et al., 2006). Tightly regulated by nitrate signaling, NRT1.1 (CHL1/NPF6.3) and NRT2.1 are most transcriptionally abundant (Wang et al., 2012). Beyond being transporters, they are also deeply involved in sensing and activating downstream gene expression, including the PNR and nitrate-regulated root development (Little et al., 2005; Remans et al., 2006a,b; Ho et al., 2009; Gojon et al., 2011).

Nitrate uptake, sensing and signaling are regulated by multiple mechanisms, which mainly include nitrate availability, feedback repression by N status, stimulation by photosynthesis, and hormone signaling (Forde, 2000; Wang et al., 2000, 2003, 2004, 2007; Nacry et al., 2013; Krouk, 2016; O’Brien et al., 2016). Significantly, primary nitrate signaling in response to nitrate availability is amplified and propagated overriding feedback constraints from downstream N metabolites and low sugars (Wang et al., 2004, 2007; Nunes-Nesi et al., 2010; Guan et al., 2017; Liu et al., 2017). The homeostasis of nitrate concentration, calcium, pH, redox, and phosphate in the cytosol is delicately regulated and maintained in all cells, for which nitrate availability is a critical determinant. For instance, the steady-state cytosolic nitrate concentrations in barley root cells were recorded between 3 and 5 mM, which corresponds with a potential “optimal” range of exogenous nitrate concentrations (1–10 mM) for plants (Miller and Smith, 1996, 2008), and cytosolic pH varies from 7.3 to 8 in plants (Martinière et al., 2013). Among the primary signaling roles of nitrate is that the disruption of cytosolic ionic environment resulting from nitrate availability/unavailability and nitrate concentrations outside of the potential “optimal” range can trigger multiple downstream cascades of N-regulated events (Champigny and Foyer, 1992; Fan et al., 2016; Liu et al., 2017; Xuan et al., 2017). The nitrate-induced disruptions are rapidly captured by the evolutionarily conserved calcium signaling. The patterns of calcium level increase are elicited by nitrate in a context-dependent manner and calcium is specifically involved in the nitrate response and signaling as a modulator and/or a second messenger (Wang et al., 2000, 2003, 2004; Riveras et al., 2015; Liu et al., 2017).

In response to low exogenous nitrate concentration (<1 mM), NRT1.1^T101^ phosphorylation is switched on, involving the CBL-interacting protein kinase CIPK23, a plant-specific calcium sensor (Ho et al., 2009; Hu et al., 2009). Through its dual-affinity binding and a phosphorylation-controlled dimerization switch between the two affinities, NRT1.1 (CHL1/NPF6.3) functions as a nitrate membrane sensor required for the PNR and other nitrate responses, independent of its uptake function as a transporter (Remans et al., 2006b; Ho et al., 2009; Wang et al., 2009). NRT1.1 (CHL1/NPF6.3) is regarded as the first transceptor discovered in plants by analogy with yeast nutrient transceptors (Ho et al., 2009; Gojon et al., 2011). It is a dose-dependent master controller of multiple signaling mechanisms capable of responding to a wide range of soil nitrate levels (Ho et al., 2009; Krouk et al., 2010a,b; Bouguyon et al., 2015; Leran et al., 2015). Nevertheless, prolonged N starvation rendered the nitrate response NRT1.1 (CHL1/NPF6.3) independent, suggesting alternative or redundant nitrate membrane sensing systems must be present (Wang et al., 2009). NRT2.1 has been proposed as such a candidate, which shows an uncoupled dual (uptake/signaling) function in root growth in response to nitrate availability (Remans et al., 2006a).

Although not capable of evoking the PNR *per se*, subgroup III calcium-dependent protein kinases (CPKs), CPK10, CPK30, and CPK32 are also required for rapid nitrate-induced cellular and metabolic responses, and nitrate-regulated root and shoot growth (Liu et al., 2017). In response to nitrate availability, Ca^2+^-sensor CPKs translocate to the nucleus, where the phosphorylation of NLP7^S205^ by CPK10 is responsible for nitrate-stimulated nuclear retention of NLP7 (Liu et al., 2017). The widely overlapped transcriptomic and phenotypic defects in *icpk* and *nlp7* mutants further substantiate the existence of the nitrate–CPK–NLP signaling-regulatory pathway potentially activating the downstream nitrate transcriptional network for signal amplification (Liu et al., 2017). Nevertheless, multiple sensing and signaling mechanisms with redundancy are synergically required for the context-dependent broad-ranged nitrate responses (Wang et al., 2009; Guan et al., 2017; Liu et al., 2017).

Furthermore, nitrate-responsive DNA regulatory elements (Girin et al., 2007; Konishi and Yanagisawa, 2010; Wang et al., 2010) and transcriptional regulators, including *ARABIDOPSIS NITRATE REGULATED 1* (*ANR1*), *NLP6, NLP7, LOB DOMAIN-CONTAINING PROTEIN 37/38/39 (LBD37/LBD38/LBD39), SQUAMOSA PROMOTER BINDING PROTEINLIKE 9 (SPL9), HIGH NITROGEN INSENSITIVE 9 (HNI9), NAC DOMAIN-CONTAINING PROTEIN 4 (NAC4), BASIC LEUCINE-ZIPPER 1 (bZIP1), TGACG MOTIF-BINDING FACTOR 1/4 (TGA1/TGA4), TCP20*, *HYPERSENSITIVE TO LOW PI-ELICITED PRIMARY ROOT SHORTENING 1 (HRS1)*, *NITRATE REGULATORY GENE2 (NRG2), BRIC-A-BRAC/TRAMTRACK/BROAD-COMPLEX 1/2 (BT1/BT2)*, and *NLP8*; (Zhang and Forde, 1998; Castaings et al., 2009; Rubin et al., 2009; Krouk et al., 2010c; Widiez et al., 2011; Konishi and Yanagisawa, 2013; Marchive et al., 2013; Alvarez et al., 2014; Guan et al., 2014, 2017; Para et al., 2014; Vidal et al., 2014b; Medici et al., 2015; Araus et al., 2016; Xu et al., 2016; Yan et al., 2016) have been identified. Some TFs, e.g., *ANR1*, *LBD37/38/39*, *SPL9*, *NAC4*, *bZIP1*, *TGA1/TGA4*, and *HRS1*, are nitrate-responsive while *NLP6, NLP7*, *HNI9, TCP20*, *NRG2*, *BT1/BT2*, and *NLP8* are not. Among the key regulators, direct interactions of NLP6, NLP7, TGA1, bZIP1, TCP20, HRS1, and NLP8 with target gene promoters have been verified. Intriguingly, multiple transcriptional mechanisms are involved in nitrate responses. bZIP1, following a hit-and-run transcriptional model, transiently bind to its target gene promoters to enable a rapid and dynamic N-signal propagation (Para et al., 2014). The propagation of nitrate signaling into metabolism and stress response are observed in the clusters of genes potentially targeted by TGA1/TGA4 (Alvarez et al., 2014). The upregulation of gene expression of *TGA1* is specifically dependent on a phospholipase C (PLC)-calcium signaling pathway downstream of NRT1.1 (CHL1/NPF6.3) (Riveras et al., 2015). In response to nitrate, NLP7 was found to be retained in nucleus via a CPK-dependent phosphorylation to promote a genome-wide gene expression regulation, giving rise to the concept of primary nitrate signaling (Castaings et al., 2009; Marchive et al., 2013; Liu et al., 2017).

**FIGURE 2:**
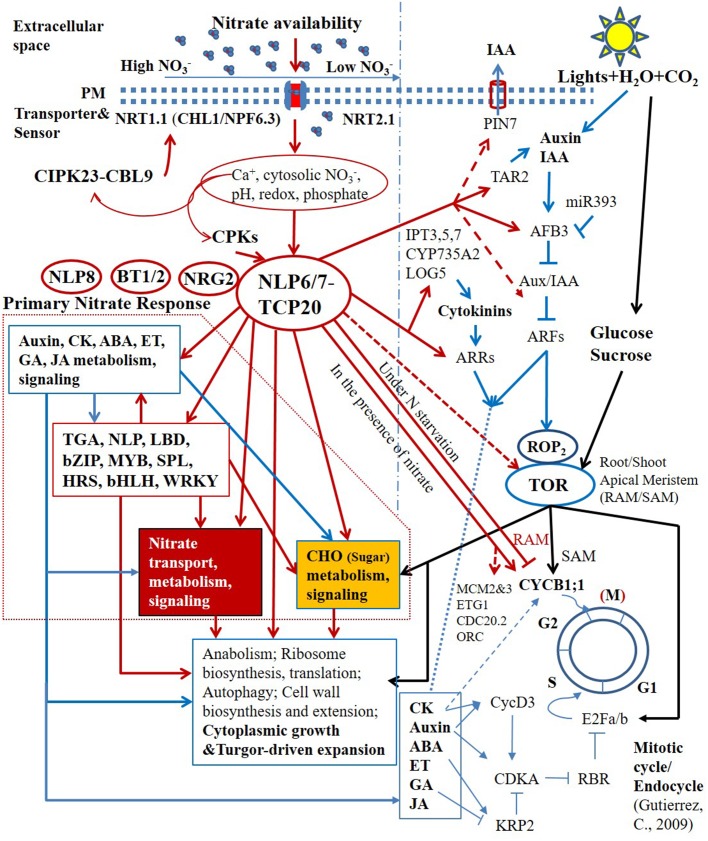
Schematic representation of the central regulatory role of TCP20-NLP6/7 complexes in nitrate transcriptional hierarchy and nutrient-growth networks. Arrows and blunted lines represent positive and inhibitory regulations, respectively. Solid lines indicate defined pathways, whereas dashed lines indicate presumed or initially confirmed pathways.

The primary nitrate signaling controlled by non-nitrate-responsive TFs that are not regulated by nitrate at the transcriptional level seems to regulate the proper level of expression of downstream nitrate-responsive TFs (**Figure 2**) (Marchive et al., 2013; Guan et al., 2014, 2017; Xu et al., 2016; Bellegarde et al., 2017; Liu et al., 2017). Moreover, several non-nitrate-responsive regulators, *NLP6, NLP7*, *TCP20*, *NRG2*, and *BT1/BT2* were shown to regulate the expression of the sentinel PNR genes, such as *NRT1.1 (CHL1/NPF6.3)*, *NRT2.1* and *NIA1* at different nitrate concentrations (Guan et al., 2014, 2017; Araus et al., 2016; Xu et al., 2016). In contrast with NLP6, NLP7, and TCP20, the specific molecular function of NRG2 is not known; nevertheless, NRG2 could be also involved in primary nitrate signaling based on its regulatory roles in the PNR and their close ties to nitrate levels (Xu et al., 2016). It is well known that the protein–protein interactions define the specificity of signal transduction and transcriptional regulation (Lamb and McKnight, 1991; Pawson and Nash, 2000). The interaction between NLP7 and NRG2 was reported; however, its function in N signaling and regulation remains to be investigated (Xu et al., 2016). Most recently, a central regulatory nexus in response to nitrate availability, involving TCP20-NLP6/7 interactions, was identified (Guan et al., 2017). Centered on this regulatory nexus, a primitive transcriptional regulatory hierarchy is emerging (**Figure 2**) (Bellegarde et al., 2017; Guan et al., 2017; Liu et al., 2017).

TCP20 and NLP6/7 proteins are constitutively and ubiquitously expressed in plants (Winter et al., 2007; Castaings et al., 2009; Hervé et al., 2009; Danisman et al., 2012; Marchive et al., 2013; Chardin et al., 2014). *TCP20* and *NLP6/7* belong to two ancient gene families, the protein sequences of which contain multiple, deeply conserved motifs in plants (Cubas et al., 1999; Schauser et al., 2005; Martín-Trillo and Cubas, 2010). The NIN-like protein gene *NLP7* was identified through its sequence similarity to the nitrate regulatory gene *NIT2* in *Chlamydomonas* (Camargo et al., 2007; Castaings et al., 2009). NLPs and RWP-RK domain proteins, whose founding members are the nodulation-specific NIN proteins, constitute the RWP-RK family. The origin of *NLPs* predates the monocot/eudicot divide. The RWP-RK proteins are key regulators of N responses in plants (Schauser et al., 2005; Chardin et al., 2014). With their origin predating the emergence of land plants, TCPs are plant-specific TFs that function as the main regulators of plant morphology and architecture, mainly because of direct transcriptional control of cell cycle genes and regulation of hormone activity by class I TCPs, (Martín-Trillo and Cubas, 2010; Manassero et al., 2013; Nicolas and Cubas, 2016). Intriguingly, distinct but overlapping binding sites between the classes I and II TCPs indicate either coordinate or competitive regulation of transcription. For example, TCP20 and TCP9 (class I) and TCP4 (class II) were found to act antagonistically on jasmonic acid (JA) metabolism and leaf development (Danisman et al., 2012). The delicate balance of transcriptional regulation between the two classes of TCPs in the distal meristem boundary zone where cell division transitions into expansion and differentiation was proposed as a key control of organ growth rate, ultimately shaping organs (Li et al., 2005). In inflorescence shoot apex, gibberellin (GA)-regulated DELLA-TCP interactions control plant height (Daviere et al., 2014).

TCP20 and NLP6/NLP7 bind to adjacent sites in the upstream promoter region of the NR gene, *NIA1*, and physically interact under continuous nitrate and N-starvation conditions. The subcellular localization and nuclear accumulation of single NLP6/7 and TCP20-NLP6/7 complexes depend on nitrate availability (Guan et al., 2017). The regulatory interactions could perceive nitrate availability via multiple upstream sensing and signal transduction in the cell membrane and cytosol, such as by the nitrate transceptor NRT1.1 (CHL1/NPF6.3), nitrate–CPK–NLP pathway (Liu et al., 2017). In the presence of nitrate, both NLP6 and NLP7 are retained in the nucleus. The nitrate-dependent nuclear retention in response to nitrate availability occurs within minutes. NLP6 and NLP7 thereby function as two partially redundant master regulators for rapid nitrate signaling and responses and growth (Marchive et al., 2013; Guan et al., 2017). The severe growth defects in *nlp6 nlp7* double mutants with nitrate as the sole N source, and in NR-null (*nia1 nia2*) mutants are comparable. Putative binding sites of NLP6 and NLP7 were found in the *CYCB1;1* promoter region. The defective *CYCB1;1* expression was also observed in *nlp6 nlp7* double mutants, which, however, did not satisfy the significance test when total roots were measured, but would likely pass the test if measuring only root tips. Under N starvation, TCP20-NLP6/7 heterodimers accumulate in the nucleus. The transcriptional complexes not only bind to and upregulate sentinel nitrate-responsive genes for transport, assimilation and signaling, but also bind to and downregulate *CYCB1;1*, a division marker of apical meristems, for the control of G2/M transition in cell cycle progression, supporting root apical meristem (RAM) growth (Li et al., 2005; Guan et al., 2014, 2017). The direct molecular link between nitrate availability and G2/M cell cycle progression in the RAM is crucial for plant adaptive postembryonic development that depends on meristems. Genome-wide transcriptional profiling further revealed that potential NLP7 targeted genes include other cell cycle genes, such as *MCM2/3*, *ETG1*, *CDC20.2*, and *ORC* (Liu et al., 2017). Therefore, TCP20-NLP6/7 complexes have a much wider presence in cell cycle regulation (**Figure 2**).

## Nitrate, Hormones, And Glucose-Tor in Root Apical Meristem Growth

TCP20 regulates mitotic cyclin gene *CYCB1;1* and putatively ribosomal protein genes by binding to the GCCCR motif in their promoters *in vitro* and *in vivo*, which was proposed to be a mechanism for regulation of the cell cycle and cell growth at a synchronized rate (Li et al., 2005; Guan et al., 2017). Under N starvation, total RNA, a majority of which is ribosomal RNA (rRNA), recorded in seedling roots of *tcp20*, *nlp6*, *nlp7*, *tcp20 nlp6*, *tcp20 nlp7*, or *nlp6 nlp7* mutants was only approximately 50% percent of the total RNA recorded in seedling roots of WT, when all were measured on same total fresh root weight, suggesting defective ribosome biogenesis and cell growth (Guan et al., 2017, unpublished results). It implies that these proteins may function as a complex in regulating ribosomal protein genes to stimulate ribosome biogenesis. The distinct nitrate-dependent TCP20-NLP6/7 interactions and their regulation under two conditions also support that nitrate signaling is an integral part of the synchronized the cell cycle and cell growth in meristem, likely via controlling cell division.

A master regulator of cytoplasmic growth is TARGET OF RAPAMYCIN (TOR), which is at the interface of growth and nutrient availability in unicellular organisms and through acquiring additional roles, becomes a central controller of organism growth, and energy and nutrient homeostasis in multicellular organisms (Zoncu et al., 2011; Sablowski and Dornelas, 2014). (m)TORC1 in both mammalian and yeast cells critically regulates and maintain the robust transcription of genes involved in ribosome biogenesis along with translation initiation and nutrient import under favorable growth conditions (Powers and Walter, 1999; Mayer et al., 2004; Xiao and Grove, 2009). That is in alignment with the roles of single NLP6 and NLP7 proteins in the presence of nitrate (Guan et al., 2017). TOR signaling is suppressed under stress conditions, leading to cell cycle arrest so as to prevent uncontrolled cell growth. However, in the face of constant nutrient stress in nature, the sustained cell growth in RAMs via transcriptionally repression of *CYCB1;1* by TCP20-NLP6/7 complexes are crucial for plants as sessile organisms. It could enable continuous nutrient acquisition in soil and adequate remobilization within plants, which are key factors for survival by maximizing NUE (Masclaux-Daubresse et al., 2010). Intriguingly, sustained RAM growth was also physiologically observed under phosphate deficiency, which is at the expense of photosynthesis (Kang et al., 2014). Moreover, during cell expansion that predominates in post-mitotic cells, the TCP20-NLP6/7 also play critical regulatory roles in cell wall biogenesis and modification (Hervé et al., 2009; Danisman et al., 2012; Karve et al., 2016). The growth of organs and whole plants depends on system-wide synchronized coordination of nutrient availability, cell growth and cell-cycle progression, for which the functions of TCP20-NLP6/7 interactions are central.

Interestingly, the TCP20-NLP6/7 regulatory nexus employs the type I/II Phox and Bem1p (PB1) domains of NLP6/7, a protein-interaction module conserved in animals, fungi, amoebas, and plants (Sumimoto et al., 2007; Chardin et al., 2014; Guan et al., 2017). In animals, PB1 domains are employed for activation of mTOR1 by amino acids and organizing growth factors (Linares et al., 2015). In plants, the type I/II PB1 domains are also employed in the homo- and hetero-oligomerization of auxin response factor (ARF) TFs and auxin/indole 3-acetic acid (Aux/IAA) repressor proteins (Boer et al., 2014; Korasick et al., 2014; Guilfoyle, 2015). This indicates that the TCP20-NLP6/7 interactions are part of a more general pattern used for nutrient–growth signaling, cellular homeostasis, and morphogenetic signaling in both plants and animals (Guan et al., 2017). However, TCP20-NLP6/7 regulatory nexus compares with recently discovered plant glucose-TOR signaling in that both of them are not well framed in conventional non-transcriptional mechanisms of mammalian TOR, which indirectly modulate limited messenger RNAs and target genes via 4E-BP1 and S6K1 phosphorylation (Xiong et al., 2013; Guan et al., 2017). Instead, they are two central transcriptional machineries controlling a broad range of nutrient–growth gene expression at the whole-plant level (Xiong et al., 2013; Guan et al., 2017).

For plant growth and development, nitrate, sugars, and the phytohormones, particularly auxin and CKs, are of vital importance. They are integral parts of the regulation of the dynamic balances of cell division and cell differentiation, which controls organ shape and size. Especially in root growth, they are intricately coordinated in controlling the balance between the cell cycle and cell growth (Xiong et al., 2013; Sablowski and Dornelas, 2014; Barrada et al., 2015; Guan et al., 2017). CK and auxin have long been implicated in regulating the components of the cell cycle (Himanen et al., 2002; Perrot-Rechenmann, 2010; Schaller et al., 2014). In *Arabidopsis*, RAM growth is under the antagonistic effects of auxin and CK, which mediate cell division at the apical meristem and cell differentiation at the transition zone, respectively (Ioio et al., 2007). For LR formation consisting of pericycle activation and meristem establishment, auxin is a dominant regulator (Himanen et al., 2002; Fukaki and Tasaka, 2009). CK was also reported to repress LR initiation and promote LR elongation (Rani Debi et al., 2005; Laplaze et al., 2007). Recently, the glucose-TOR signaling pathway was reported to control the G1/S transition by an unconventional mechanism of transcriptional regulation. TOR kinase directly phosphorylates and activates E2Fa, which in turn transcriptionally activates S-phase genes in response to glucose and sucrose signaling, which is independent of S6K, RBR or translational control (Xiong et al., 2013). The glucose-TOR signaling for the glycolysis-mitochondrial energy relays is indispensable for RAM growth.

Intriguingly, the induction level of primary auxin and CK marker genes and spatial expression of patterning genes were intact in the presence of rapamycin or antimycin A (AMA) in WT (in both cases TOR activity is inhibited) and in *tor* seedlings. Upon glucose starvation, neither RAM cell number nor RAM length were significantly reduced, which is overtly opposite to what was observed in RAM upon N starvation, where arrest at the G2/M transition occurred (Xiong et al., 2013; Guan et al., 2017). Under N starvation, significant reductions of LR number per plant were displayed across the mutant lines, *nlp6*, *nlp7*, *tcp20 nlp6*, *tcp20 nlp7*, and *nlp6 nlp7* (Guan et al., 2017, unpublished results). It is consistent with that in *Arabidopsis*, the initial xylem pole pericycle cell divisions during first LR initiation event are accompanied with regulation of G2/M transition (Himanen et al., 2004; Malamy, 2005). Auxin and CK signaling and stem cell niche maintenance seems not to rely on sugar signaling and metabolism pathways (Xiong et al., 2013). The accumulating evidence as reviewed here suggests that nitrate signaling and metabolism is crucial for hormone signaling and maintenance of stem cell niche integrity.

TORC1 and TORC2 complexes, and a large part of the evolutionary “core” of TOR pathway likely originated in or before the last eukaryotic common ancestor (LECA) that gave rise to all currently known living eukaryotic species (van Dam et al., 2011). Although the two TOR complexes are found in other major lineages of eukaryotes, plants possess only TORC1 (van Dam et al., 2011). In plant TOR signaling as has been uncovered so far, the integration of N status with cell growth and the cell cycle, which is the main component in TOR1 pathways of both yeast and mammals and required by virtually all eukaryotic cells, is still missing. It is mainly because the upstream regulators that directly sense N availability and organize growth factors are unknown. Plants lack orthologs of small guanosine 59-triphosphatases (GTPases): Ras homolog enriched in brain (RHEB), and Rag guanosine 59-triphosphatases (RAGs) (Xiong and Sheen, 2014). TOR signaling is highly conserved; however, it is adequately flexible to include new signals and mechanisms in response to environmental challenges in the evolution of animals and plants (van Dam et al., 2011). For example, insulin signaling is an animal-specific addition to the pathway to use sugar (glucose) for cellular growth in a multicellular environment. Recently, the plant-specific small GTPase Rho-related protein 2 (ROP2) (Li et al., 2001) was demonstrated to transduce light-auxin signal to activate TOR by direct interaction (Li et al., 2017). Interestingly, TOR kinase can also be activated by nitrate and amino acids via S6K1 T449 phosphorylation by unknown mechanisms in *Arabidopsis* seedlings (Xiong and Sheen, 2015). Could TCP20-NLP6/7 complexes function in parallel with plant TOR or upstream of plant TOR for N signaling?

The glucose-TOR signaling imposes an overall limit on plant organ growth, specifically on organ size (Sablowski and Dornelas, 2014). By contrast, nitrate and its close interplay with hormones have a determining effect on patterning tissues and shaping organs (Crawford, 1995; Stitt, 1999; Forde, 2002; Miller et al., 2008; Ruffel et al., 2011; Nacry et al., 2013; Guan et al., 2014, 2017; Mounier et al., 2014; Sablowski and Dornelas, 2014; Krouk, 2016; Ristova et al., 2016). This is a strong indication of interaction and integration of nitrate and hormonal signaling pathways in plant growth and development. One thing is clear: RAM growth that takes place underground is particularly under convergent regulation of nitrate and hormone signaling. Notably, the distinct mechanism and coordination between hormone and glucose-TOR energy signaling are involved in regulation of shoot apical meristem (SAM). Light, a main aboveground environmental cue, is required for producing auxin, which in turn can activate downstream ROP2-TOR-E2Fa/b signaling pathway and promoting SAM growth (Li et al., 2017). The involvement of nitrate signaling in SAM growth should also be investigated in the integrated context.

## Nitrate in Auxin Biosynthesis, Transport, Signaling, And Responses

Auxins are a class of essential phytohormones involved in tailoring plant growth and morphology to environmental conditions (Vanneste and Friml, 2009; Overvoorde et al., 2010). As the main endogenous auxin in most plants, indole-3-acetic acid (IAA) is the most potent native auxin, regulating almost every aspect of plant life, i.e., growth, development, and biotic and abiotic stress responses (Woodward and Bartel, 2005; Zhao, 2012). IAA biosynthesis is defined by a two-step complete pathway where indole-3-pyruvate (IPA) is converted from tryptophan (Trp) by the TAA family of amino transferases, before IPA being converted to IAA by the YUC family of flavin monooxygenases (Zhao, 2012). It is generally accepted that auxin regulation of plant morphogenesis relies on its tissue-specific concentration gradients collectively formed by the processes of auxin biosynthesis, conjugation, degradation and transport (Normanly, 2010). Recent studies also showed that localized auxin biosynthesis is indispensable in many developmental processes including embryogenesis, seedling growth, root development, vascular patterning, phyllotaxis and flower development (Cheng et al., 2006, 2007; Overvoorde et al., 2010; Pinon et al., 2013; Chen et al., 2014). In *Arabidopsis* roots, defective localized auxin biosynthesis cannot be replenished by auxin transported from shoots, indicating that shoot-derived auxin alone is not sufficient for supporting root elongation and root gravitropic responses (Chen et al., 2014).

Notably, N availability directly regulates *TAA1* and its close homologs *TAR1* and *TAR2* in the first step of IAA biosynthesis (Ma et al., 2014). In *Arabidopsis*, the expression levels of the three genes in roots and shoots under high N conditions (3 mM NH_4_NO_3_) were compared with those under low N conditions (0.1 mM NH_4_NO_3_) after 7 days treatment (Ma et al., 2014). The expression of *TAR2* was significantly induced by low N in roots, where the expression of *TAA1* was moderately induced. The expression of *TAA1* and *TAR1* were both repressed in shoots. *TAR2* was expressed in the root pericycle and vasculature of root maturation zone near the root tip. The *tar2* mutants showed repressed auxin accumulation in LR primordia and reduced LR primordia emergence and numbers under low N conditions (Ma et al., 2014).

However, with ammonium in the media, it was difficult to identify which N source, nitrate or ammonium, or both of them, could be responsible for the gene expression regulation. The recent genome-wide transcriptional profiling showed that *TAR2* and *PIN-FORMED PROTEIN 7* (*PIN7*) are among the top NLP7-activated genes, together with nitrate assimilation genes such as *NiR*, *NIA1*, *FNR2*, and *NRT2.1* (Marchive et al., 2013; O’Malley et al., 2016; Liu et al., 2017). Plasma membrane-localized PIN7 is a main auxin efflux carrier protein (Friml et al., 2003). This evidence substantiates that the auxin biosynthesis and transport is transcriptionally regulated by nitrate in roots. It further suggests that the TCP20-NLP6/7 complexes function upstream of auxin-ROP2-TOR-E2Fa/b signaling pathway (**Figure 2**).

Auxin transport has been postulated to be a major factor determining intercellular and intracellular distributions of IAA. In plant cells, transporters and their asymmetrical localization are required for suggested directional efflux of anionic auxins and formation of polar flow. Therefore, NLP7-regulated auxin efflux via PIN7 could contribute to regional auxin gradient and local maxima to establish and maintain a root primordium and determine LR numbers (Overvoorde et al., 2010). Auxin was also shown to be transported away from the LR primordium by NRT1.1 (CHL1/NPF6.3) at low nitrate conditions (<0.5 mM), therefore preventing the growth of pre-emerged LR primordia and young LRs; when nitrate being plentifully supplied, auxin accumulated in the LR primordium to promote growth as a result of repressed auxin transport activities of NRT1.1 (CHL1/NPF6.3) (Krouk et al., 2010b; Mounier et al., 2014). The phosphorylated form of NRT1.1 (CHL1/NPF6.3) is predominantly active in auxin transport among the point mutations in NRT1.1 (CHL1/NPF6.3) being tested for auxin influx activity in *Xenopus oocytes*. It is also responsible for modulation of auxin gradient in LR primordium (Bouguyon et al., 2015).

Transport of other plant hormones across plasma membranes also requires transporter proteins that are spatiotemporally regulated during development instead of occurring simply by diffusion (Saito et al., 2015). Interestingly, more new substrates, such as ABA, GAs, jasmonoyl-L-isoleucine, and glucosinolates, were recently found to be transported by NRT1/PTRs or NPFs (Kanno et al., 2012; Nour-Eldin et al., 2012; von Wittgenstein et al., 2014; Saito et al., 2015; Chiba et al., 2015; Tal et al., 2016). In addition to being nitrate transporters, the capability of transporting hormones was suggested to be another critical feature of this family in plants. However, no long-distance transport of any hormones, i.e., loading/unloading of them into/out of xylem/phloem vessels, has been demonstrated. All the NRTs-dependent transports so far reported only involve local redistribution of the hormones (Kanno et al., 2012; Tal et al., 2016).

In *Arabidopsis* roots, a miR393/AFB3 regulatory module was identified as nitrate-responsive, which integrates nitrate and auxin signaling in modulating both primary and LR growth (Vidal et al., 2010). *miR393* was the only N-responding sRNA identified in 454 sequencing and it specifically responded to nitrate not sucrose. The auxin receptor genes, *TIR1*, *AFB1*, *AFB2*, and *AFB3*, are regulated by miR393. Among them, a strong induction of *AFB3*, also the only induction, by nitrate, was observed. The *AFB3* induction peaked at 1 h after nitrate (5 mM KNO_3_) exposure, and the nitrate induction of miR393 peaked at 2 h, strikingly coinciding with the fast declining of the already peaked expression of *AFB3*. This miR393-dependent repression was not observed in NR-null mutants, correlating with the absence of *miR393* expression. Further evidence support that nitrate signal alone is responsible for the transcriptional induction of *AFB3* in root tips, which can be subsequently post-transcriptionally repressed by miR393 induced by unidentified N metabolite(s) downstream of nitrate reduction. Such a mechanism agrees with the type I incoherent feed-forward loop (FFL) motif featured in transcriptional controls in yeast, bacteria, and mammals (Shen-Orr et al., 2002; Mangan and Alon, 2003; Tsang et al., 2007; Vidal et al., 2010). Accompanied with it, accumulation of auxin and the regulatory of many auxin-responsive and auxin-related genes involved in multi-level of auxin signaling and responses in the root tips and pericycle cells were also observed. The nitrate-regulated miR393/AFB3 module is capable to integrate nitrate (5 mM) signal into auxin-dependent root growth. Shorter primary root due to inhibited root elongation and more dense LRs due to higher rate of LR initiation and emergence were formed in response to nitrate availability in soil (Vidal et al., 2010).

Auxin binds to TIR1/AFB receptors, members of the SCF^TIR1/AFB^ E3 ubiquitin ligase complex. It promotes the recognition and degradation of the Aux/IAA repressors via by polyubiquitination, which free the inhibition of the auxin response factors (ARFs) that allows auxin-responsive transcription (Chapman and Estelle, 2009). The activation of *AFB3* is not the cause but one of the consequences of nitrate response (Vidal et al., 2013). AFB3-dependent auxin signaling, including perception and response, and its regulation of root growth is downstream of nitrate signaling in response to nitrate availability, independent of nitrate transport and metabolism. Specifically, *NAM/ATAF/CUC* TF, *NAC4* and its targeted TF gene *OBP4*, functions as a downstream branch of nitrate-*AFB3.* The nitrate-*AFB3*-*NAC4*-*OBP4* signaling, with all their proteins found expressed in root pericycle cells, is required for nitrate-dependent LR initiation and emergence. The *NAC4*-*OBP4* part of the pathway is possibly regulated by AUX/IAA proteins, such as IAA14. These observations suggest convergent regulation between nitrate and auxin signaling pathways on LR growth (Vidal et al., 2013).

With three different NRT1.1 (CHL1/NPF6.3) mutants: *chl1-5*, *chl1-9*, and NRT1.1^T101D^ mutants, the role of nitrate membrane sensor/transporter, NRT1.1 (CHL1/NPF6.3) in regulating nitrate response of *AFB3* and *NAC4* were tested (Vidal et al., 2014a). Specifically, *chl1-5* is a deletion mutant without uptake and sensing function; *chl1-9* is defective in both high- and low-affinity nitrate uptake but not in nitrate signaling, and NRT1.1^T101D^ mutant mimics a constitutively phosphorylated transporter, with only the high-affinity mode (Ho et al., 2009). Interestingly, not like sentinel PNR genes, such as *NRT2.1*, *NIA1*, and *NIA2*, which were tightly controlled by the signaling functions of NRT1.1 (CHL1/NPF6.3), only the transport function of the NRT1.1 (CHL1/NPF6.3), not those of NRT2.1, NRT1.2 (NPF4.6/AIT1), and NRT2.2, matters on the expression levels of *AFB3* and *NAC4*. Moreover, the nitrate induction of *AFB3* and *NAC4* was independent of affinity mode of NRT1.1 (CHL1/NPF6.3). Notably, the NRT1.1^T101D^ was demonstrated to modulate auxin gradient in LRP as WT NRT1.1 (CHL1/NPF6.3) in the absence of nitrate, which excludes the possible involvement of auxin transport of NRT1.1 (CHL1/NPF6.3) in the nitrate induction of *AFB3* and *NAC4* (under 5 mM nitrate treatment). It was suggested that an unidentified signaling pathway independent from the signaling via NRT1.1 (CHL1/NPF6.3) phosphorylation was triggered by NRT1.1 (CHL1/NPF6.3) transport of nitrate (Vidal et al., 2014a). It further substantiates that nitrate responses, which include the nitrate-*AFB3*-*NAC4*-*OBP4* auxin perception, signaling and response, are established via multiple signaling mechanisms and their coordination (Liu et al., 2017).

## Nitrate in Cytokinin Biosynthesis, Signaling, And Responses

Involved in many phases of plant growth and development, cytokinins (CKs) are a class of phytohormones known for promoting cell division and differentiation (Mok and Mok, 2001). CKs can interact with auxins either synergistically or antagonistically and also promote the production of ethylene (Mok and Mok, 2001). Since CKs are translocated at cellular and whole-plant levels, CK root-shoot communication is proposed as a model of systemic signaling for nutrient status (Sakakibara, 2006; Ruffel et al., 2011). CK activity in plants is tightly related to nitrate availability. Nitrate, not its downstream N metabolites, has been known to induce rapid *de novo* CK synthesis and accumulation in the roots of barley, maize, and *Arabidopsis* (Takei et al., 2004). CK biosynthesis can also occur in other tissues where the *adenosine phosphate-isopentenyltransferases* (*IPTs*) are expressed. IPTs are key enzymes that catalyze the first and rate-limiting step of CK biosynthesis, i.e., prenylation of adenosine 5′ phosphates, such as ATP and ADP, at the N^6^-terminus with dimethylallyl diphosphate (DMAPP) (Sakakibara, 2005). *IPT* expression is ubiquitous and peaks in proliferating tissues. In *Arabidopsis*, *IPT3* is regulated by N in a nitrate-specific manner. The expression of *IPT3* and several *Arabidopsis response regulators 3, 5, 6* (*ARR3, 5, 6*), is induced by nitrate during the PNR (Wang et al., 2004). *IPT3* was strongly induced in roots and weakly induced in shoots in both WT and NR-null mutant plants during the PNR, partly mediated by NRT1.1 (CHL1/NPF6.3) (Wang et al., 2004, 2009). When nitrate (10 mM KNO_3_) was re-supplied to nitrogen-limited seedlings, the kinetics of *IPT3* and *NIA1* transcripts that were rapidly accumulated within 1 h. resembled each other (Takei et al., 2004). *NIA1* is among the most induced genes in the PNR (Wang et al., 2000); therefore, nitrate has a tight control over CK biosynthesis via activation of *IPT3*. All are consistent with the idea that IPT3 is the main determinant of short-term nitrate-dependent CK biosynthesis, particularly in roots, in response to the rapid change of nitrate availability in soil (Takei et al., 2004). More recent transcript profiling of CK metabolism and signaling genes further revealed that besides *IPT3*, high nitrate upregulates the transcripts of *CYP735A2*, which is responsible for the production of *trans*-zeatin-type (*t*Z-type) CK in roots, while it downregulates that of *LOG5*. The type-A ARR genes *ARR3*, *ARR5*, and *ARR7*, like the CK metabolism genes, were shown to respond to nitrate but not to ammonium (Ramireddy et al., 2014; Liu et al., 2017). Also induced by nitrate are *CYTOKININ RESPONSE FACTORS (CRFs)* (Rashotte et al., 2006; Liu et al., 2017), which is known to be transcriptionally induced by CK and whose disruption affects the basal expression of a significant number of CK-regulated genes, including the type-A ARRs. CRFs are implicated in promoting root and shoot growth and leaf senescence (Raines et al., 2016).

Among the most highly expressed *IPTs*, *IPT3* is mainly expressed in phloem tissue throughout the whole plant, specifically found in phloem companion cells, and *IPT5* is in the LR primordium and pericycle, which are consistent with where CK biosynthesis is suggested to occur. The spatial differentiation of *IPTs* transcript also suggests that in terms of CK production, IPT5 and IPT3 could contribute most in roots, while IPT3 is most dominant in shoots. *IPT5* was not responsive to either nitrate or ammonium under a short-term hour-long treatment, however, was demonstrated to be a “long-term” or systemic N status-responsive gene, with its transcript abundance being responsive to both nitrate and ammonium media at different concentrations over a long course of observation, 11 days. By contrast, *IPT3* expression pattern (strongly in roots and weakly in shoots) is quickly induced during the PNR, a frequently occurring whole-plant nitrate response due to dramatic fluctuations of nitrate in soil (Wang et al., 2004). The close regulation of *CYP735A2* and *IPT3* by nitrate could be a major factor shaping nitrate-dependent spatiotemporal CK distribution in plants and regulating root system architecture in response to a variety of abiotic stresses (Ramireddy et al., 2014).

Compared to the rapid nitrate response of *IPT3* (within 1 h), CK signaling has a relatively delayed (within 4 h) feedback control over most the *IPTs, IPT1*,*3,5,7* by downregulating them in roots, where *IPT5* and *IPT7* can be upregulated concurrently by auxin (Miyawaki et al., 2004). *IPT7* is expressed in root stele and phloem companion cells. To add another layer of dynamic complexity of interactions, CK and auxin also exert feedback controls over nitrate uptake and assimilation (Guo et al., 2002; Krouk, 2016). In this context, nitrate and two hormonal mediators, CK and its antagonistic partner, auxin, act in concert to modulate CK biosynthesis in root development. The dual nitrate-CK response system, employing *IPT3* and *IPT5*, along with CK/auxin feedback regulations on *IPTs* could have a critical role in mediating root foraging for nitrate, a classic plant response to nitrate availability. To compete for nutrients in diverse soil microenvironments, plants have evolved the unique capability to proliferate LRs preferentially in nutrient-rich zones, called “root foraging” (Drew et al., 1973). Root foraging for nitrate involves both local and systemic signaling (Ruffel et al., 2011; Guan et al., 2014; Mounier et al., 2014). Besides their effects on localized CK and auxin biosynthesis, the concerted nitrate-CK-auxin regulation could also be an integral part of N systemic signaling that coordinates nutritional requirements among different organs and at different developmental stages. Notably, ammonium and downstream N metabolites are unlikely to be major players in systemic N signaling (Howitt and Udvardi, 1999; Forde, 2002; Bellegarde et al., 2017).

Using WT, NR-null and *ipt3,5,7* mutants in split-root experiments, nitrate signaling was demonstrated to act both locally and systemically to integrate N supply and demand. The systemic N signaling also involves a nitrate-CK relay, where *IPT3, IPT5 and IPT7* play a central role. The nitrate-CK relay is suggested to be necessary for shaping root foraging (Ruffel et al., 2011). The study also suggested that there is an additional systemic signaling pathway also required. Using decapitation experiments, the concept of shoot-root CK-dependent feedback specifically for N demand was proposed. However, questions remain. Is decapitation a definitive way to confirm CK’s independence from nitrate in the systemic N demand signaling? In analogy to CK’s root-shoot-root signaling/relay mechanism, a similar model was proposed for small peptides in root foraging (Okamoto et al., 2013; Tabata et al., 2014; Ohkubo et al., 2017). Clavata3/ESR (CLE)-related peptide signal and N starvation-triggered C-terminally Encoded Peptide (CEP) were identified as “satiety” and “hunger” signals. In root-to-shoot route, CLE and CEP were first derived from roots, then transmitted to shoots where being perceived by leucine-rich repeat receptor-like kinase (LRR-RLK) receptors HAR1 and CEPRs, respectively. In the following shoot-to-root route, CEP Downstream1 (CEPD1) and CEPD2, two phloem-specific polypeptides, are regulated by CEPRs and then transmitted to roots, where NRT2.1 is thereby activated (Ohkubo et al., 2017). Notably, the two hormone-dependent systemic signaling pathways could be necessary but not sufficient for root foraging independent of local and systemic signaling by nitrate (Ruffel et al., 2011, 2016; Guan et al., 2014; Mounier et al., 2014).

Furthermore, there is also intriguing spatiotemporal regulation of CK signaling by nitrate in the context of root foraging. The expression of primary CK-response genes and negative regulators of CK signaling, type-A *ARRs(3,5-9)* was globally up-regulated by nitrate in roots and shoots at a later time (2 h, 8 h, and 2 days) compared with the much quicker expression of *IPT* (within 1 h) (Ruffel et al., 2011). The response levels of *ARRs* in NR-null roots and shoots are very comparable, in some cases even lower in roots, which is strikingly opposite to those of *IPTs* (Wang et al., 2004). Since both CK biosynthesis after IPT induction and induction of *ARRs* by the produced CK are rapid (Wang et al., 2002), the much later (>8 h) regulations of *ARRs* by nitrate (Ruffel et al., 2011) suggest additional nitrate-regulated mechanism(s) are likely to be involved rather than CK biosynthesis-dependent replay.

Another branch of the evidence that deserves our attention is that TCP20 as a cell-autonomous systemic nitrate regulator is clearly required for the nitrate foraging by roots (Guan et al., 2014). *tcp20* mutants strongly suppress the preferential growth of LRs by equalizing growth across heterogeneous nitrate environments, mainly through increasing the LR growth on low-nitrate media of split-root plates as if the plants were impervious to any systemic signal. An earlier study showed that the main class of AtTCP20::EAR-repressed genes include *ARR4,6,7* and *AUX/IAA13,16,27* that repress ARFs in auxin signaling (Hervé et al., 2009). All the genes possess at least one class I TCP binding motif in their promoters. In *tcp20* mutants, the foraging-defective LR growth in high/low nitrate media was largely due to much shorter/longer LR length but not to the less/more number of LRs (Guan et al., 2014). The RAM growth is indeed under the balanced control of nitrate-CK-auxin signaling as also previously discussed. The concerted nitrate-CK-auxin signaling could also have TCP20 as a mediator between CK and auxin for the regulation of root foraging.

## Nitrate in Abscisic Acid Deconjugation, Degradation, Transport, and Signaling

Nitrate sensing, signaling and regulation, and their interaction with hormones are very dose-dependent. Beyond the optimal range (1–10 mM) corresponding to steady-state cytosolic nitrate concentrations (4–6 mM) (Miller and Smith, 2008), additional interaction between nitrate and hormones occurs. Transferring *Arabidopsis* seedlings between media with different nitrate concentrations has been used to mimic plant responses to a variety of nitrate availability in soil. The reversible oscillating responses have been thereby observed in nitrate-dependent hormone biosynthesis and accumulation, and LR growth (De Smet et al., 2003; Tian et al., 2009).

Abscisic acid has been long regarded as a stress hormone crucial to plant abiotic and biotic stress responses (Zhu, 2002; Kiba et al., 2012). In the face of high nitrate concentrations (approximately >10 mM), nitrate and ABA are close partners, especially in the control of LR growth (Signora et al., 2001; De Smet et al., 2003). Nitrate serves as an osmolyte; therefore, the changes of nitrate availability and concentration alter osmotic potential of plant cells. Repression of LRs in *Arabidopsis* due to very high nitrate (30 mM) resembled the repressed growth of LR treated by 30 mM KCl or 60 mM mannitol. All the treatments, including high nitrate (>30 mM), could impose osmotic stress (Deak and Malamy, 2005). Exogenous ABA also inhibits LR development, mimicking high nitrate repression of LR (De Smet et al., 2003). Furthermore, ABA synthesis and ABA-sensing mutants displayed significantly reduced inhibitory effects by high nitrate concentrations (>10 mM) (Signora et al., 2001). The ABA-induced growth arrest occurred right after LR emergence and before the activation of the LR meristem, which is due to ABA suppression of the transcription of two cell cycle-related genes, *CYCD3;1* and *CDKB1;1*. It serves as a checkpoint that, however, is reversible (De Smet et al., 2003).

The accumulation of ABA was detected mainly in the endodermis and quiescent center of *Arabidopsis* root tips, similar to the expression pattern of *SCARECROW*, and to a lesser extent in the vascular cylinder (Ondzighi-Assoume et al., 2016). A threefold increase of ABA level in root tips was observed in seedlings being transferred from the medium containing 20 mM nitrate to that containing 30 mM nitrate. It was accompanied by the increased activity of the endoplasmic reticulum-localized, ABA-GE-deconjugating enzyme β-GLUCOSIDASE1, but not with *de novo* ABA biosynthesis. High nitrate thereby stimulates release of bioactive ABA from the inactive storage form, ABA-glucose ester (ABA-GE) (Ondzighi-Assoume et al., 2016). In parallel with this, osmotic stress causes accumulation of the endogenous ABA; therefore, ABA has been regarded as a mediator of responses of osmotic stress imposed by drought and high salt (Zhu, 2002). All suggests that both ABA and high nitrate share a single pathway which is likely part of general osmotic stress responses, during a specific LR development stage in *Arabidopsis*. And notably the high nitrate-ABA pathway is independent of auxin (De Smet et al., 2003).

ABA-IMPORTING TRANSPORTER (AIT) 1, also characterized as the constitutive, low-affinity nitrate transporter NRT1.2 (NPF4.6), mediates cellular ABA uptakes during seed germination and post-germination growth of *Arabidopsis* (Kanno et al., 2012). In response to drought stress, plants synthesize ABA to trigger closing of stomatal pores. NRT1.2 (NPF4.6/AIT1) is suggested to be involved in regulation of stomatal aperture in inflorescence stems via transporting ABA synthesized in vascular tissues to guard cells. Being an osmolyte, nitrate is also known for controlling gas exchange by stomates. In the presence of nitrate, NRT1.1 (CHL1/NPF6.3) is required in nitrate induced depolarization and nitrate accumulation in guard cells during stomatal opening. Its mutants showed reduced stomatal opening and transpiration rates in the light or when deprived of CO_2_ in the dark, leading to drought resistance (Guo et al., 2003). The two nitrate transporters seem to be able to work in a “coordinated” manner to regulate the stomatal functions in response to drought stress or nitrate, whichever signal becomes dominant. An intriguing coordination between the two transporters occurred when the induction of NRT1.1 (CHL1/NPF6.3) by nitrate caused a transient repression of NRT1.2 (NPF4.6/AIT1) (Huang et al., 1999). With the exception of this temporary coupled reaction of NRT1.1 (CHL1/NPF6.3) and NRT1.2 (NPF4.6/AIT1) in response to nitrate induction, *NRT1.2 (NPF4.6/AIT1)* is constitutively expressed before and after nitrate exposure (Huang et al., 1999).

The mechanism of interaction between nitrate and ABA signaling, which could be behind such coordination is further understood in roots. In another study, ABA insensitive2 (ABI2; an ABA inactivated PP2C) has been identified as a potential interacting protein of the CBL1-CIPK23 complex, which like CBL9-CIPK23 has inhibitory effects on nitrate transport of NRT1.1 (CHL1/NPF6.3), under >30 mM nitrate (Leran et al., 2015). The CBL9-CIPK23 complex is known to be responsible for the phosphorylation of the NRT1.1 (CHL1/NPF6.3), resulting in switching to high-affinity transport mode in response to low nitrate availability (<1 mM) (Ho et al., 2009). ABI2 negatively regulates the full activation of CBL1-CIPK23 toward their targeted proteins by substantially reducing CIPK autophosphorylation and CIPK-dependent phosphorylation of the Ca^2+^-sensor moiety in the associated CBL (Hashimoto et al., 2012). During drought and osmotic stresses, stress-induced ABA could inactivate ABI2 by RCAR/PYL/PYR interaction, which enhances phosphorylation of NRT1.1 (CHL1/NPF6.3) and phosphorylation of AKT1 by CBL1-CIPK23. The similar phenotypes associated with nitrate transport and signaling were observed in both *chl1* and *abi2-2* mutants. The mechanism could allow plants to rechannel their energy and resource from nitrate assimilation to stress response via reducing nitrate uptake in favor of uptake of potassium ions, which is critical in abiotic and biotic stress responses (Wang et al., 2013). It suggests that ABA-dependent stress signals could be required to be conveyed to and processed through the nitrate transceptor, NRT1.1 (CHL1/NPF6.3), so that the abiotic stress response is likely a collective decision made in conjunction with nitrate signaling. The conclusion is also supported by the results of an earlier study that in *Arabidopsis* guard cells, ABI1 and ABI2 protein phosphatases are downstream of NR-mediated nitric oxide (NO) in the ABA signal-transduction cascade (Desikan et al., 2002). The NO synthesis regulated by nitrate signaling is required for ABA-induced stomatal closure (Desikan et al., 2002).

Recently, a direct molecular link between nitrate signaling and ABA degradation in seed germination was revealed (Yan et al., 2016). The conserved nitrate regulator, NLP8, was found to regulate ABA catabolism and activate the expression of *CYP707A2*, which is indispensable for nitrate-induced seed germination. This activation appears to occur directly, through NLP8 binding to the promoter of *CYP707A2*, which encodes ABA 8′-hydroxylase, a key ABA catabolic enzyme (Kushiro et al., 2004; Yan et al., 2016). ABA negatively regulates the germination process. Hence, seed germination after the onset of imbibition can be triggered in a timely fashion upon reduced level of ABA. Notably, CYP707A2 has been shown to be a hub processing environmental signaling, i.e., nitrate, light, and temperature, during germination (Footitt et al., 2011, 2013).

## Nitrate in Ethylene Biosynthesis and Signaling

With the chemically simplest form among phytohormones, ethylene is a gaseous signal molecule and potent regulator of developmental adaptations (Ecker, 1995; Bleecker and Kende, 2000). The production of ethylene is regulated by internal signals during developmental phases, including seed germination, root growth, fruit ripening, organ senescence, etc., and also in response to biotic and abiotic stresses (Wang et al., 2002). Compared with the significant ABA accumulation in roots of the seedlings that were transferred from low nitrate (20 mM) to high nitrate (30 mM), transferring seedlings from low nitrate (0.1 mM) to high nitrate (10 mM) caused a rapid burst of ethylene production in roots (Tian et al., 2009). Both of them contribute to the inhibitory effects of LR growth exerted by transferring to high nitrate conditions. Strikingly, the LR growth inhibition and the elicited ethylene evolution can be reversed by transferring the seedlings back to the low nitrate (0.1 mM), similar to the reversible arrest observed in the case of ABA (De Smet et al., 2003). Ethylene is synthesized from methionine through *S*-adenosyl-L-methionine and 1-aminocyclopropane-1-carboxylic acid (ACC), which are catalyzed by ACC synthase (ACS) and ACC oxidase (ACO) (Kende, 1993). The nitrate-dependent ethylene evolution and accumulation were accompanied by transient but significant increase of ACS and ACO, which are transcriptionally induced by the transferring to high nitrate (10 mM). The inactivation of ACS and ACO by their antagonists alleviated LR growth defects. Nitrate-induced ethylene inhibited the growth of immature LRs, which is at a later development stage compared to ABA-induced LR inhibition.

Employing the combinations of *Chl1-5* and *nrt2.1-1* mutants and ethylene-insensitive mutants, *etr1-3* and *ein2-1*, ethylene was demonstrated as an important modulator in the regulation of nitrate-dependent expression of the two main transporters, NRT1.1 (CHL1/NPF6.3) and NRT2.1 (Tian et al., 2009). Notably, in the comparable range of low nitrate (0.5 mM), NRT2.1 promotes initiation of LR primordia, which is likely a different mechanism occurring at an early stage of LR development (Remans et al., 2006b). The ethylene-dependent regulation, or nitrate signaling relay (Krouk, 2016) observed here when seeding roots being challenged by high nitrate conditions, could be centered on NRT1.1 (CHL1/NPF6.3) whose expression is much more strongly affected. This signaling relay via ethylene might be part of the mechanism where NRT1.1 (CHL1/NPF6.3) mediates the repression of NRT2.1 between high-affinity transport mode and low-affinity transport mode (Muños et al., 2004; Krouk et al., 2006). Interestingly, transferring seedlings from high nitrate (10 mM) to low nitrate (0.1 mM) also caused a rapid burst of ethylene production measured on a whole-plant basis. NRT2.1 whose repression is relieved by NRT1.1 (CHL1/NPF6.3) under low nitrate concentration (<0.5 mM) was singled out to be responsible for stimulating ethylene production (Zheng et al., 2013).

## Nitrate in Gibberellin Biosynthesis, Transport, And Signaling

Gibberellins are tetracyclic diterpenoid hormones. GAs are key endogenous regulators involved in seed germination, root and shoot elongation, flowering, and fruit patterning (Daviere et al., 2014; Tal et al., 2016). Much of the lead role of nitrate in dancing with hormones has been revealed in roots. Nevertheless, nitrate-hormone interaction certainly takes place in whole plants. For example, in the transition from vegetative growth to reproduction, earlier flowering was favored at low nitrate growth conditions rather than at high nitrate conditions. The major repressor of flowering in *Arabidopsis*, *FLOWERING LOCUS C* (*FLC*), is repressed and activators of flowering, *FLOWERING LOCUS T* (*FT*), *LEAFY* (*LFY*), and *APETALA1* (*AP1*), are induced in low-nitrate conditions. Interacting with photoperiod- and temperature- and GA-signaling pathways, nitrate regulates floral induction by communicating nutrient availability (Castro-Marín et al., 2011; Kant et al., 2011; Liu et al., 2013). The low nitrate (1 mM) was shown to transcriptionally induce expression of *GA1*, the main GA biosynthesis gene, therefore promoting bioactive GAs in various tissues of flowering plants. Along with *GA1*, nitrate also induced the expression of *SUPPRESSOR OF OVEREXPRESSION OF CO 1 (SOC1)*, an integrator of the GA-dependent flowering pathway which coordinates all the endogenous pathways: GA, vernalization, autonomous, and photoperiod (Liu et al., 2013). The transcriptome of pre-starved *Arabidopsis* seedlings in response to nitrate re-addition (3 or 5 mM KNO_3_) also showed the repressed expression of *GID1B*, GA receptor and the induced expression of *GATA, NITRATE-INDUCIBLE, CARBON-METABOLISM INVOLVED (GNC)* and *GNC-LIKE/CYTOKININ-RESPONSIVE GATA FACTOR1* (*GNL/CGA1)* TFs, negative regulators of GA signaling downstream from DELLA proteins and PHYTOCHROME-INTERACTING FACTORS (PIFs) (Wang et al., 2003, 2004; Scheible et al., 2004; Richter et al., 2010).

*NPF3.1* expression in endodermis were found to be transcriptionally repressed by GA and promoted by ABA (Tal et al., 2016). *NPF3.1* is a plasma membrane localized protein mediating nitrate and nitrite uptake (Sugiura et al., 2007; Leran et al., 2014). In addition, the experiments in *X. oocytes* showed that NPF3.1 is an active GA importer and it is also capable of transporting ABA. Another NPF protein, AIT3/NPF4.1, was found earlier to have ABA and GA transport activities (Kanno et al., 2012). NPF3.1 as such, is involved in two antagonistic hormone signaling in endodermal cells controlling root meristem size (Tal et al., 2016). Such an intimate interplay between nitrate, GA and ABA were also observed in determining seed dormancy and germination times (Alboresi et al., 2005; Chopin et al., 2007; Yan et al., 2016). GTR1/NPF2.10 was also proposed as a multifunctional transporter employed by the structurally distinct compounds glucosinolates, JA-Ile and GA, which promote stamen development via mediating bioactive GA transport (Saito et al., 2015).

## Conclusion

Decades of nitrate research have given rise to new paradigms. By analogy with molecular oxygen (O_2_) being an environmental morphogen in embryonic development and stem cell function in animals (Simon and Keith, 2008), and auxin being proposed as a plant morphogen (Bhalerao and Bennett, 2003; Esmon et al., 2006), nitrate could be a potent environmental morphogen in plants given the comprehensive nitrate transport, sensing, signaling and regulations at the level of the cell and organism (Wang et al., 2004, 2012; Bellegarde et al., 2017). Remarkably, less than 0.1% seed N is from nitrate (Chopin et al., 2007), which doesn’t prevent nitrate from being a crucial signal and creating specific niches (Yan et al., 2016); and phloem-based transport of nitrate that represents only 1–10% of total N in the phloem sap, has a more morphogenetic role than a nutritional role in sink organs (Fan et al., 2009; Kiba et al., 2012; Hsu and Tsay, 2013; Bellegarde et al., 2017). The differential responses to extracellular nitrate availability while maintaining cellular homeostasis, especially steady-state ionic environment, are among the main morphogenetic effects in determining cell growth and identity in plants. At a more detailed level, the intercellular and intracellular gradients of nitrate could be responsible for diversified patterns of transient and sustained promotion or repression of expression levels of specific subsets of nitrate-responsive genes, which were observed in a variety of nitrate responses, including the PNR. Behind the whole-plant responses is that the changes of N status of the shoot could be potentially communicated to the root via the peaks and valleys of nitrate gradient and xylem- and phloem-based transport, serving for long-distance signaling (Bellegarde et al., 2017).

Why does nitrate signaling critically regulate so many types of phytohormones at so many levels? The localized hormone biosynthesis, deconjugation and degradation seem to be the primary connection between nitrate and hormones, for which solid molecular evidence has been increasingly found. Being an environmental cue and once being absorbed into plants, also becoming an environmental morphogen, nitrate transcriptionally regulates the metabolism and signaling of hormones at the whole plant level. Indeed, this regulatory process is highly context-dependent and spatiotemporal. Conversely, the hormones from nitrate-induced production, deconjugation and degradation, could act as mediators and/or modulators in N-dependent signaling and regulation and provide feedback controls at the regional level. Notably, numerous hormone signaling and regulatory components were found to be transcriptionally activated by nitrate in certain contexts, suggesting that the signaling of the two morphogens is intertwined at multiple levels, if not all levels. The genome-wide transcriptome further revealed that the molecular conductors at the top-level of nitrate regulatory hierarchy could exert direct controls over hormonal pathways (Hervé et al., 2009; Danisman et al., 2012; Marchive et al., 2013; Guan et al., 2017; Liu et al., 2017), so that a variety of hormones are employed in propagation and amplification of nitrate signaling (**Figure 2**).

Intriguingly, cell-autonomous regulation by N in determining cell growth and fate is strongly indicated by TCP20-NLP6/7 regulatory nexus, which is involved in sensing nutrient status and transcriptional control of G2/M transition in cell cycle progression (Guan et al., 2017). Growing evidence suggests that there could exist PB1 domain-mediated interactions between nitrate and auxin signaling regulators upstream of TOR signaling, which are central in nutrient–growth process in plants (Xiong et al., 2013; Guan et al., 2017; Li et al., 2017). Moreover, the analogy between regulatory roles of TCP20 in response to nitrate availability and of TCP21/CHE in the circadian oscillator suggest a general TCP-dependent cell-autonomous mechanism for plant responses to variations in environmental cues, i.e., nutrients, light, and temperature (Pruneda-Paz et al., 2009; Guan et al., 2017). The intertwined coordination of cell autonomous and morphogen-gradient-dependent mechanisms is deeply conserved in eukaryotes, being well observed in the amoeba, *Dictyostelium discoideum* (Clay et al., 1995).

Hormones have long been regarded to provide an indispensable link between N and plant growth and development. However, among the most deeply conserved in plants, nitrate signaling and regulation with a highly organized transcriptional hierarchy are as crucial as hormonal signaling and regulation in growth, development and stress responses. The novel model that underlies substantial plant development and adaptive responses could involve other TCPs because of functional redundancy between TCP20 and its homologs. The classes I and II TCPs exert either coordinate or competitive regulation of transcription that could be essential for defining growth rate and organ development (Li et al., 2005). The interaction between TCPs and hormone biosynthesis, transport, signaling and responses in growth, development and defense has been increasingly reported (Nicolas and Cubas, 2016). With TCPs in the picture, much extended interplay and convergent regulation between nitrate and hormone signaling will be expected.

In the past half century, N fertilizer is the main contributor to global crop production increases that support two billion more people on Earth. Nevertheless, NUE has become a major constraint on agricultural productivity and environmental sustainability worldwide. Understanding nitrate signaling and regulation and their interaction with hormones is central to meet the global challenges, which demands extensive research under the new paradigms.

## Author Contributions

The author confirms being the sole contributor of this work and approved it for publication.

## Conflict of Interest Statement

The author declares that the research was conducted in the absence of any commercial or financial relationships that could be construed as a potential conflict of interest. The reviewer EW and handling Editor declared their shared affiliation.
